# Mechanistic Understanding of Curcumin’s Therapeutic Effects in Lung Cancer

**DOI:** 10.3390/nu11122989

**Published:** 2019-12-06

**Authors:** Wan Nur Baitty Wan Mohd Tajuddin, Nordin H. Lajis, Faridah Abas, Iekhsan Othman, Rakesh Naidu

**Affiliations:** 1 Jeffrey Cheah School of Medicine and Health Sciences, Monash University Malaysia, Jalan Lagoon Selatan, Bandar Sunway, Selangor Darul Ehsan 47500, Malaysia; wan.wanmohdtajuddin@monash.edu (W.N.B.W.M.T.); iekhsan.othman@monash.edu (I.O.); 2 Laboratory of Natural Products, Faculty of Science, Universiti Putra Malaysia, UPM, Serdang 43400, Malaysia; nordinlajis@gmail.com (N.H.L.); faridah@food.upm.edu.my (F.A.); 3 Department of Food Science, Faculty of Food Science and Technology, Universiti Putra Malaysia, UPM, Serdang 43400, Malaysia

**Keywords:** lung cancer, curcumin, anti-cancer, molecular mechanism

## Abstract

Lung cancer is among the most common cancers with a high mortality rate worldwide. Despite the significant advances in diagnostic and therapeutic approaches, lung cancer prognoses and survival rates remain poor due to late diagnosis, drug resistance, and adverse effects. Therefore, new intervention therapies, such as the use of natural compounds with decreased toxicities, have been considered in lung cancer therapy. Curcumin, a natural occurring polyphenol derived from turmeric (*Curcuma longa*) has been studied extensively in recent years for its therapeutic effects. It has been shown that curcumin demonstrates anti-cancer effects in lung cancer through various mechanisms, including inhibition of cell proliferation, invasion, and metastasis, induction of apoptosis, epigenetic alterations, and regulation of microRNA expression. Several in vitro and in vivo studies have shown that these mechanisms are modulated by multiple molecular targets such as STAT3, EGFR, FOXO3a, TGF-β, eIF2α, COX-2, Bcl-2, PI3KAkt/mTOR, ROS, Fas/FasL, Cdc42, E-cadherin, MMPs, and adiponectin. In addition, limitations, strategies to overcome curcumin bioavailability, and potential side effects as well as clinical trials were also reviewed.

## 1. Introduction

Lung cancer is a highly malignant tumor and one of the most common types of cancer worldwide. In 2012, it was estimated that 1.8 million new lung cancer cases represent approximately 12.9% of all cancers diagnosed worldwide [1]. According to the estimation of The International Agency for Research on Cancer (IARC), there was 2.1 million new lung cancer cases and the number of lung cancer-associated death rose to 1.8 million worldwide in 2018 [2]. 

Lung cancer is classified into two types which include non-small cell lung cancer (NSCLC) and small cell lung cancer (SCLC) that account for approximately 80% and 20% of all lung cancer cases respectively [3]. Due to the limitations of effective screening and asymptomatic clinical manifestation at early stage of disease, 70%–80% lung cancer patients were diagnosed at an advanced stage [4]. To date, the standard treatments for the majority of lung cancer patients including SCLC and NSCLC are surgery, chemotherapy, radiation therapy, or a combination of these treatments [5]. In addition, targeted therapy drugs such as tyrosine kinase inhibitors (TKIs) of epidermal growth factor receptor (EGFR) and anaplastic lymphoma kinase (ALK) are used to treat NSCLC patients [6]. In recent years, immunotherapy drugs have been introduced to treat some patients of NSCLC by targeting the programmed cell death receptor on T cells [7]. Unfortunately, all of the standard treatments, targeted therapy, and immunotherapy drugs have been reported to cause several side effects and toxicities in lung cancer patients [5,8,9]. Furthermore, the prognosis of lung cancer remains poor as the five-year survival rate for all stages combined is approximately 16% despite the significant advances in diagnostic and therapeutic approaches [10]. It has been indicated that poor prognosis is associated with late diagnosis, drug resistance, and toxicity [11]. Therefore, further investigations and research on lung cancer mechanisms, non-toxic therapeutics drugs, and new intervention targets are significant as the understanding in this context would prove useful in lung cancer therapy. 

More than 35,000 plants species including spices and herbs are being consumed extensively around the world as they are believed to have numerous therapeutic properties with reduced side effects [12]. Among these species, curcuma longa (also known as turmeric) has been used widely since ancient times as a spice in traditional cuisine as well as a traditional medicine in Southeast Asia, India, and China [13]. Curcumin which is the main component of turmeric has been shown to have numerous therapeutic activities including anti-inflammatory [14], anti-oxidant [15], anti-microbial [16], anti-atherosclerosis [17,18] and anti-cancer [19]. Multiple studies have revealed that curcumin may act as a potential chemopreventive agent as well as a novel adjuvant treatment for cancer [20]. In addition, clinical trials have proven curcumin is a safe and well tolerated dietary constituent for humans, and with anti-carcinogenic capability [21]. Several studies in vitro and in vivo showed curcumin demonstrates anti-cancer effects on lung cancer through mechanisms such as inhibition of cell proliferation, induction of apoptosis, epigenetics changes, and regulation of microRNAs expression [20]. In this current review, modulation of multiple molecular targets and signaling pathways including STAT3, EGFR, FOXO3a, TGF-β, eIF2α, COX-2, Bcl-2, PI3KAkt/mTOR, ROS, Fas/FasL, Cdc42, E-cadherin, MMPs, and adiponectin were discussed. In addition, strategies to overcome curcumin bioavailability, limitations, and potential side effects, as well as clinical trials were also reviewed.

## 2. Mechanism of Anti-Cancer Effect on Lung Cancer 

### 2.1. Effects of Cell Proliferation and Cell Cycle

Cell proliferation results in increased number of cells and highly regulated in normal cells. It is defined by coordinated cell cycle that creates the balance between cell division and cell loss. Therefore, dysregulation of the cell cycle can cause excessive or uncontrolled proliferation, contributing to the development of malignant tumor cells [22,23]. A number of studies have documented curcumin mediated anti-proliferative effect in lung cancer cells via modulation of various molecular targets such as STAT-3, EGFR, Forkhead Box O3 (FOXO3a), Eukaryotic Initiation Factors (eIFs), and Transforming Growth Factor-Beta (TGF-β). 

#### 2.1.1. Signal Transducer and Activator of Transcription 3 (STAT3)

STAT3 protein is a member of the STAT protein family that serves as a transcription factor transmitting signals from the cell surface to the nucleus [24]. STAT3 is activated through STAT3 tyrosine phosphorylation by Janus Kinase (JAK) and growth factor receptor tyrosine kinase in response to the bonds of cell surface receptors to ligands such as epidermal growth factor (EGF), interferons and interleukin-6 (IL-6) [25,26]. Activation of STAT3 is a feature in normal human cells as it involves in regulation of multiple cellular functions such as cell proliferation, differentiation, host defense, and development [27]. While activated STAT3 protein in normal cells is well controlled and has a short lifespan, the activated STAT3 protein in cancer cells including lung cancer is continuously active leading to uncontrolled proliferation, apoptosis resistance, as well as sustained angiogenesis [28,29,30]. It has been reported that activated STAT3 protein is expressed in over 50% of NSCLC primary tumors and cell lines [31,32,33] while 100% of SCLC tumor tissues tested contain high level of activated STAT3 protein [34]. Persistent activation of STAT3 protein has been correlated with enhanced cell proliferation in both NSCLC through its ability to induce the expression of several growth-promoting genes such as *c-myc*, *Pim-1*, and *cyclin D1* [35,36,37].

Recently, researchers found that suppression of STAT3 phosphorylation has contributed to anti-proliferative effect of curcumin against both SCLC and NSCLC. Yang et al. (2012) revealed that curcumin inhibits the STAT3 phosphyorylation in SCLCNCI-H446 and NCI-1688 which in turn causes downregulation of cyclin B1, a key component in the control of cell cycle progression from G2 to M phase. Thus, this activity subsequently arrests the G2 phase of cell cycle and inhibits the cell proliferation. In addition, this study also showed that the inhibition of STAT3 phosphorylation by curcumin is able to cause loss of colony formation, and the inhibit migration and invasion of cancer cells [38]. Similarly, Wu (2015) showed that curcumin inhibited the JAK2/STAT3 signaling pathway in NSCLC NCI-H460 cells, which resulted in downregulation of *cyclin D1* and *c-myc* that serve as regulators of cell cycle progression and transcriptional factor respectively. Consequently, it leads to the inhibition of cell proliferation and colony formation in NCI-H460 lung cancer cells. Curcumin could also reduce tumor spheres of NCI-H460 cells by inhibiting the JAK2/STAT3 signaling pathway in both in vitro as well as in vivo [39]. The anti-proliferative effect of curcumin on lung cancer cells via the STAT3 phosphorylation pathway has been further confirmed in both in vitro and in vivo studies by Alexandrow et al. (2012). It was indicated that human lung adenocarcinoma H441 cells are sensitive to curcumin exposure in a dose-dependent manner resulting in a reduction of cell proliferation. In agreement with this, the results also showed that curcumin suppresses STAT3 phosphorylation activity and further inhibits expression of cyclin D1 and mcm2 markers indicating a reduced proliferative ability [40]. In a recent study, Tang et al. (2018) demonstrated that curcumin may act as a STAT3 inhibitor to inhibit proliferation in lung squamous cell carcinoma NCI-H292. The results revealed that inhibition of STAT3 increased Forkhead box transcription factor A2 (FOXA2) expression, and it has been reported that overexpression of FOXA2 reduced cell growth, inhibited cell proliferation, and induced apoptosis as it functions in regulating the expression of genes critical to lung morphogenesis [41]. Taken together, the above findings suggest that the anti-proliferative effect of curcumin via the STAT3 signaling pathway could be a potential target for lung cancer chemotherapeutic therapy.

#### 2.1.2. Epidermal Growth Factor Receptor (EGFR) 

EGFR also known as HER-1 or ERbB1 belongs to the family of growth factor receptor tyrosine kinases (TKs). It has been described previously that EGFR-TKs are involved in fundamental cellular functions such as cell proliferation, division, and differentiation [42,43]. The activation of EGFR results in activation of Raf/MEK/Erk, STAT, and P13k/AKT pathways which lead to cell survival [44,45,46]. Multiple evidence suggests that aberrant EGFR expression and signaling contribute to tumorigenesis as well as progression of various cancer types including lung cancer [47,48,49]. Elevated expression of EGFR is found in 62% of NSCLC and it is associated with poor prognosis as well as reduced survival rate in lung cancer patients [50,51,52]. It has been demonstrated that curcumin downregulates EGFR in multiple cancer cells including NSCLC and subsequently inhibits its cell proliferation. A study by Jiang and co-investigators (2014) revealed that curcumin downregulated EGFR in A549 lung cancer cells and increased expression of *UBE1L*. *UBE1L* has been regarded as a potential tumor suppressor gene and decreases overall levels of cyclin D1. Hence, this promotes the suppression of cell growth [53]. 

#### 2.1.3. Forkhead Box O3 (FOXO3a)

FOXO3a belongs to a family of the Forkhead box class O (FOXO) transcription factor which plays a crucial role in cellular functions such as cell cycle arrest, apoptosis, DNA damage repair, and differentiation, as well as stress detoxification [54,55]. Several studies demonstrated that a reduced level of FOXO3a expression contributes to cell transformation, tumor progression, and angiogenesis in a variety of cancer cells including lung cancer [56,57,58]. Several studies have reported that curcumin mediates anti-cancer effect in cancer cells including neuroblastoma and lung cancer through modulation of FOXO3a expression. It has been demonstrated that curcumin and its analogues increase the expression of FOXO3a in A549 and H460 human lung cancer cells through enhancement of ROS production, subsequently elevating the expression of FOXO3a target genes including *p21*, *p27* and *Bim* while decreasing the level of cyclin D1. Of note, both inhibitor protein p21 and p27 suppresses G1 to S cell cycle transition, followed by cell proliferation inhibition of A549 and H460 cells. In addition, this study also showed that curcumin suppresses the growth of A549 lung cancer xenograft tumors associated with suppression of proliferation in tumor tissues [59].

#### 2.1.4. Transforming Growth Factor Beta (TGF–β)

Transforming growth factor-β belongs to a family of three multifunctional cytokines that plays an important role in the regulation of cell proliferation as well as differentiation in most human epithelial tissues [60]. TGF-β signaling regulates several cellular functions such as cell growth, differentiation, angiogenesis, apoptosis, and extracellular matrix remodeling [61]. Deregulation of TGF-β expression has been associated with tumor development and progression depending on tumor types and stages [62]. Previous studies have demonstrated that TGF-β exhibits anti-tumor roles in NSCLC through the smad pathway. Consequently, it activates TGF-β responsive gene expression, suppresses cell proliferation, induces apoptosis as well as reduces tumorigenicity [63,64,65]. Curcumin has been reported to regulate TGF-β signaling cascade in neonatal lung fibroblast [66], renal cells [67], keloid fibroblasts [68], and scleroderma fibroblasts [69]. Interestingly, Datta and colleagues (2013) revealed that the anti-cancer effect of curcumin on NSCLC cells in vitro and in vivo on mouse tumor xenograft was linked irrespectively to the TGF-β/Smad signaling pathway. The findings showed that curcumin has no significant effect on both TGF-β sensitive and TGF-β insensitive NSCLC cell lines. Hence, it can be suggested that curcumin could be a potential anti-cancer agent for both TGF-β sensitive as well as TGF-β resistant NSCLC tumors [70].

#### 2.1.5. Eukaryotic Initiation Factors 2 Alpha (eIF2α)

The eIF2α is a regulatory subunit of eIF2α which plays a pivotal role to initiate translation of protein synthesis process in eukaryotic cells. Notably, phosphorylation of eIF2α results in inactivation of the whole eIF2 and reduces the level of active eIF2α inhibiting global protein synthesis [71]. Previous studies have revealed that a higher level of eIF2α has been observed in oncogene transformed cells and tumor cells such as Hodgkin’s lymphoma [72], esophageal cancer [73], gastrointestinal carcinomas [74], and malignant melanoma [75], as well as bronchioloalveolar carcinomas of the lung [76]. Increased expression of eIF2α leads to increased protein synthesis, which is associated with tumorigenesis. Chen and co-investigators demonstrated that curcumin inhibits cell proliferation as well as cell viability of A549 lung adenocarcinoma cells through modulation of eIF2α expression. Curcumin decreases the expression of eIF2α and increases the phosphorylation of eIF2α which inhibits initiation of translation and inhibition of protein synthesis, thus suppressing cell proliferation [77].

**Figure 1:**
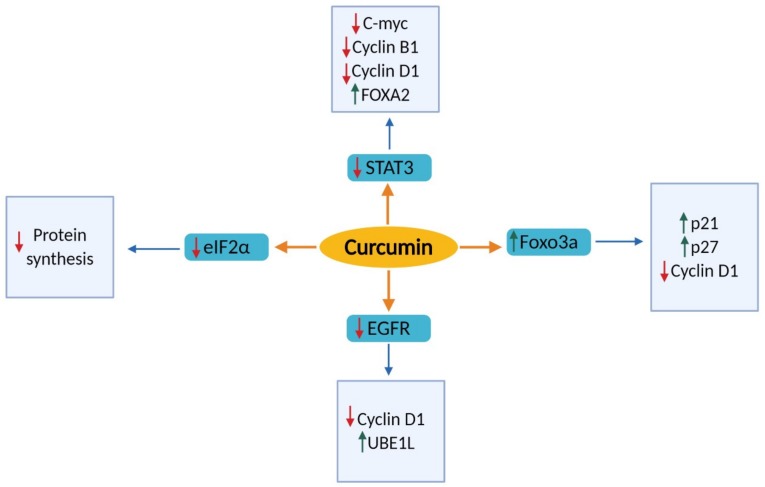
Molecular targets of curcumin in inhibiting cell proliferation of lung cancer cells. The green arrow indicates upregulation, while the red arrow indicates downregulation of molecular targets. STAT 3: Signal transducer and activator of Transcription 3; eIF2α: Eukaryotic initiation factors 2 alpha; EGFR: Epidermal growth factor receptor; Foxo3a: Forkhead box class O; FOXA2; Forkhead box transcription factor A2; *UBE1l*: Ubiquitin-like modifier-activating enzyme; C-myc: C-myc proto-oncogene.

The above findings indicate that the anti-proliferative effects of curcumin against lung cancer are associated with the modulation of transcription factors, protein kinases, and cell cycle regulatory proteins as presented in Figure 1.

### 2.2. Effects on Apoptosis

Apoptosis or programmed cell death, has been known as an essential and highly regulated event in cell homeostasis and eukaryotic development. Deregulation in apoptotic process can result in the tumor cell formation as it creates a permissive environment for genetic pathway instability and the accumulation of mutations [78]. Thus, making the molecular pathways of apoptosis as the most potent targets to counter the cancerous growth. Curcumin has been shown to induce apoptosis in lung cancer cells via both intrinsic and extrinsic pathways by regulating multiple molecular targets including Cyclooxygenase 2 (COX-2), Bcl-2 family, reactive oxygen species (ROS), death receptors, and signaling pathways such as Phosphatidylinositol-3-kinase (PI3K).

#### 2.2.1. Cyclooxygenase 2 (COX-2)

Cyclooxygenase also referred as prostaglandin H synthase-2 or PTGS2 is an enzyme that responsible for the production of postanoids including prostaglandins and thromboxanes from free arachidonic acid [79]. Accumulating evidences have shown frequent upregulation of COX-2 expression in both pre-malignant and malignant tissues including lung cancer, suggesting that COX-2 is one of the key factors in carcinogenesis [80,81,82,83,84,85]. The elevated expression of COX-2 was found to impact multiple pathways involved in malignant progression such as resistance to apoptosis, promotion of angiogenesis, increased proliferation, as well as increased malignancy [86,87,88]. Higher expression of COX-2 level has been observed in at least 70% of lung adenocarcinomas and squamous cell carcinomas compared to the adjacent normal lung tissue [89]. It has been shown that inhibition of COX-2 able to induce apoptosis in COX-2 overexpressing lung cancer cells [90]. 

Curcumin has been revealed to serve as a potential COX-2 inhibitor in multiple types of cancer including lung cancer, for its ability to suppress COX-2 expression. In a study with NSCLC P14 cells, curcumin downregulates COX-2 and EGFR expressions. This study showed that the COX-2 product, prostaglandin E_2_ (PGE_2_), is able to transactivate the EGFR pathway through four G protein-coupled receptors (GPCRs), resulting in the promotion of cancer cell growth and motility. The cross-talk between the EGFR signaling and COX-2 pathways was associated with a decreased extra-cellular signal regulated kinase (ERK1/2) activity which resulted in the inhibition of cell survival and induction of apoptosis [91]. In addition, curcumin on COX-2 downregulation is mediated through NF-κB. Curcumin has been shown to inhibit activation of NF-κB that suppresses IKK which inhibits phosphorylation and degradation of IkB-kinase alpha (IkBa) followed by p65 nuclear translocation [92]. A study by Charalambous and co-workers found that upregulation of COX-2 was accompanied by elevated expression of NF-κB-p65 and IkBa in human colorectal cancer epithelial cells [93]. Similarly, as reported in a recent study on NSCLC H1975 cells, curcumin inhibits COX-2 expression through modulation of NF-κB, IkBa and p65 expression which in turn increased induction of apoptosis and reduced survival of the NSCLC cells. Furthermore, in vivo experiments also demonstrated that curcumin caused 36% reduction in weight of intra lung tumors and downregulation of COX-2 expression [94].

#### 2.2.2. B-cell Lymphoma-2 (Bcl-2) Family Member

Bcl-2 family proteins are key regulators of apoptosis through mitochondrial apoptotic pathways by promoting caspase cascade activation [95]. Bcl-2 family members are classified into two groups which include anti-apoptotic proteins (Bcl-2, Bcl-X_L_, Bcl-W) and pro-apoptotic proteins (Bax, Bak, Bcl-Xs, Bad, Bid) [96]. The balanced ratio of various Bcl-2 family members play a vital role in cellular apoptotic homeostasis. It has been reported that, elevated expression of Bcl-2 has been observed in numerous types of cancer including lung cancer [97]. Bcl-2 overexpression was found in 19%–33% of NSCLC cases [98,99,100], while up to 90% of SCLC cases [101,102]. The overexpression of Bcl-2 in lung cancer cases has been linked to poor prognosis as well as cell survival. 

Wu and co-workers (2010) reported that curcumin treatment downregulates Bcl-2 and Bcl-XL but upregulates Bax and Bad proteins in NSCLC NCI-H460 cells. The ratio between pro- and anti-apoptotic Bcl-2 family members on mitochondrial membranes was displaced leading to increased membrane permeability followed by leakage of cytochrome C into the cytosol [103]. Consequently, these changes caused activation of caspase cascade and induced apoptosis [104,105]. Similarly, curcumin induced apoptosis in NSCLC A549 cells through the regulation of Bcl-2/Bax protein that affects the mitochondrial apoptotic pathway [106,107]. In addition, another study has revealed that curcumin induced apoptosis in NSCLC cells by continuous elevation of Ca^2+^ that was caused by downregulation of Bcl-2 protein [108]. It has been reported that Bcl-2 suppression plays an important role in the regulation of calcium release from endoplasmic reticulum (ER) through IP3R phosphorylation. Bcl-2 acts as a docking protein to facilitate the interaction of IP3R and calcineurin, which then dephosphorylates IP3R, decreasing the channel activity [109]. Excessive accumulation of Ca^2+^ in the cytoplasm leads to the opening of mitochondrial permeability transition pore (mPTP) as well as enhances mitochondrial outer membrane permeabilization (MOMP) followed by releasing cytochrome C into the cytosol [110]. These findings suggest that the downregulation of Bcl-2 by curcumin in NSCLC cells is associated with calcium overload which contributes to mitochondrial-dependent apoptosis. 

#### 2.2.3. Phosphatidylinositol-3-kinase-Akt-mTOR (PI3K/Akt/mTOR)

Phosphatidylinositol-3-kinase (PI3K), a lipid kinase family, is activated by the binding of extracellular growth factors to transmembrane receptor tyrosine kinases (RTKs) such as the vascular endothelial growth factor receptor (VEGFR), EGFR, insulin-like growth factor 1 receptor (IGF-1R), fibroblast growth factor receptor, and others [111]. Functional PI3K is recruited to the plasma membrane converting phosphatidylinositol (4,5)-bisphosphate (PIP_2_) to phosphatidylinositol (3,4,5)-triphosphate (PIP_3_). Subsequently, PIP_3_ localizes Akt to the plasma membrane and binds to the pleckstrin homology (PH) domain of Akt/Pkb which leads to activation of Akt [112]. Activated Akt plays an important role in the phosphorylation and inhibition of downstream signaling proteins such as Bcl-associated death promoter (Bad), Bax, Glycogen Synthase Kinase 3 (GSK3), and FOXO transcription factors thereby initiating cell cycle progress and inhibiting apoptotic signals. Moreover, Akt also indirectly activates mTOR which is involved in cell growth and metabolism via phosphorylation and inhibition of tuberous sclerosis complex 1/2 (TSC 1/2) [113,114]. As a result, activation of these targets contributes to elevation of cell proliferation, metabolism, growth, and survival. Accumulating evidences have shown that PI3K/Akt/mTOR pathway is deregulated in lung cancer and has been associated with high-grade tumors, advanced disease, and poor prognosis [114,115]. Alteration of PI3K/Akt/mTOR pathway was found in 50%–70% of NSCLC cases [114,116] and approximately 36% of SCLC cases [117]. 

Curcumin was shown to inhibit PI3K/Akt/mTOR pathways in NSCLC cells A549 [118] and H1299 [119]. The findings demonstrated that curcumin treatment suppresses the PI3K/Akt/mTOR pathway by decreasing the Akt and mTOR phosphorylation thus inducing apoptosis [119,120]. On a different note, a tumor suppressor phosphatase and tensin homolog (PTEN) protein has been found to inactivate PI3K/Akt/mTOR pathways through dephosphorylation of PIP_3_ to PIP_2_ [121]. Reduction or absence of PTEN has been observed in 30% to 70% of NSCLC [122,123] and 8% to 40% of SCLC [124,125,126]. Curcumin also has been shown to increase PTEN expression via modulation of miR-21 in NSCLC cells A549 which leads to activation of PI3K/Akt/mTOR pathway followed by induction of apoptosis [127]. 

#### 2.2.4. Reactive Oxygen Species (ROS)

Reactive oxygen species (ROS) are highly reactive radicals, ions, or molecules that contain unpaired electrons and are formed by the partial reduction of molecular oxygen [128]. It is well documented that elevated levels of ROS has been found in multiple types of cancers including lung cancer cells. Accumulation of ROS leads to uncontrolled cell proliferation, angiogenesis, metastasis, and resistance to apoptosis in cancer cells [129]. 

Several studies have been demonstrated that curcumin exhibits apoptosis in multiple cancer cells including lung cancer through ROS or oxidative stress signaling pathway. Previous studies on SCLC NCI-H446 [130] and NSCLC A549 [131] cells treated with curcumin showed induction of apoptosis via reactive oxygen species-mediated mitochondrial pathway. The findings in these studies found that curcumin increased Bax expression but downregulated Bcl-2 and Bcl-X_L_ expression, leading to a decrease in mitochondrial membrane potential. Subsequently, cytochrome C release in combination with an apoptotic protease-activating factor (Apaf-1) contributes to the formation of a complex known as apoptosome, which activates caspase-9 followed by caspase-3 [130,131]. 

Another study conducted by Yao and co-workers demonstrated that HSP70 was involved upon elevation of ROS production by NSCLC A549 cells treated with curcumin followed by induction of apoptosis. HSP70 is expressed highly under stress conditions, such as heat shock as well as oxidative stress. It has been indicated that HSP70 was highly expressed in tumor cells and associated with histological types of lung cancer and prognosis. High levels of HSP70 activate the Bcl-2 protein family and inactivate caspase 9, thus causing inactivation of caspase 9, which affects the cytoplasm of the caspase cascade and inhibits apoptosis. Curcumin has been found to suppress the activity of HSP70 via intracellular redox state regulation, and as a result, the mitochondrial apoptotic pathway was activated. [132]. In addition, it has been demonstrated that curcumin induces apoptosis in NSCLC A549 cells via ROS and mitogen-activated protein kinase (MAPK) signaling pathways [131,132]. The MAPK pathways regulated by ROS are closely associated with cell proliferation and differentiation as well as necrosis. MAPK pathway includes 3 major kinases which are ERK, JNK, and P38. The ERK kinase plays a role in cell differentiation and proliferation [133], and meanwhile JNK and P38 are involved in the regulation of cell apoptosis [134,135]. In this study, phosphorylated JNK and p38 proteins were increased in response to curcumin, whereas ERK was reduced in a dose-dependent manner. The results showed that curcumin induces apoptosis in A549 cells through the activation of MAPK signaling pathway, and expression of p38, JNK, and ERK proteins [132,136].

#### 2.2.5. Fas–Fas Ligand interactions

Fas (CD95 or APO-1) serves as a cell surface receptor which its role is pivotal for apoptotic signaling in different types of cells [137,138]. The Fas receptor binds with FasL, a natural ligand that belongs to a member of the tumor necrosis factor superfamily to initiate the death signal cascade, hence resulting in apoptosis via extrinsic and intrinsic pathways [139,140]. The Fas receptor has been found widely in numerous type of tissues, while FasL expression is commonly found in immune system cells such as activated T cells and natural killer cells, and the cells within immune privileged areas, such as the reproductive organs and eyes [141,142,143]. Nonetheless, decreased expression of Fas and/or increased expression of FasL has been detected in multiple types of human cancer, including lung cancer, and appears to be a feature of the malignant phenotype, suggesting that the Fas/FasL system may play an important role in cancer formation [144,145]. There is a strong evidence demonstrating that decreased expression of Fas may protect transformed cells from elimination by anti-tumor immune responses, but heightened expression of FasL may increase the ability of tumor cells to counterattack the immune system by killing Fas sensitive lymphocytes and therefore contribute to lung cancer development [146,147,148,149,150]. Curcumin has been reported to increase Fas/CD95 expression as well as caspase-8 activity in NCI-H460 cells suggesting that extrinsic apoptotic pathway was activated following the treatment. These findings were further confirmed by a significant increase in cell viability after pre-treatment of curcumin-treated NCI-H460 cells with caspase-8 inhibitor [103]. 

**Figure 2:**
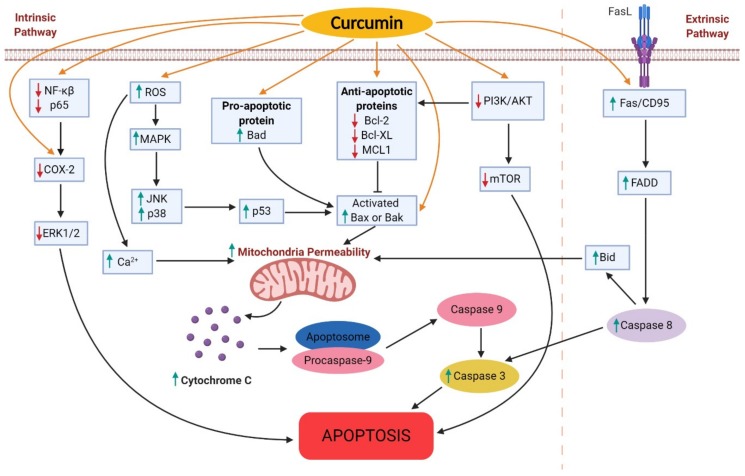
Molecular targets of curcumin in inducing cell apoptosis of lung cancer cells. The green arrow indicates upregulation, while the red arrow indicates downregulation of molecular targets. NF-κB: nuclear factor kappa-light-chain-enhancer of activated B cells; COX-2: cyclooxygenase-2; ERK1/2: extra-cellular signal regulated kinase: ROS: reactive oxygen species; MAPK: mitogen-activated protein kinase; JNK: Jun N-terminal kinase; PI3K: phosphatidylinositol 3-kinase; Akt: protein kinase; mTOR: mammalian target of rapamycin; Bad: Bcl-2-associated death promoter; Bcl-2: B-cell lymphoma-2; BCL-xL: B-cell lymphoma-extra large; MCL1: induced myeloid leukemia cell differentiation protein; FADD: Fas-associated protein with death domain.

As summarized in Figure 2, curcumin induces apoptosis through the modulation of numerous molecular targets that are involved in intrinsic and extrinsic pathways of apoptosis. Thus, it can be suggested that targeting the molecular pathways of apoptosis is one of the effective approaches in lung cancer therapy.

### 2.3. Effects on Cell Invasion and Metastasis

Tumor metastasis is a multistep event which includes alteration in various biochemical, genetic, and epigenetic factors in the primary tumor that contributes to the invasion–metastasis cascade [151]. 

It has been shown that curcumin inhibits metastasis in lung cancer cells by modulating several molecular targets including Cdc42, E-Cadherin, matrix metalloproteinases (MMPs), VEGF, and adiponectin.

#### 2.3.1. Cell Division Cycle 42 (Cdc42)

Cell division cycle 42 (Cdc42) is a member of Rho family of GTPases, which serves as an important molecular switch converting an inactive GDP-bound form to an active GTP-bound form in response to diverse signals. The activation of Cdc42 is regulated by guanine nucleotide exchange factors (GEFs), GTPase activating proteins (GAPs) and guanine nucleotide dissociation inhibitors (GDIs) [152]. Activated Cdc42 involves in many cellular processes such as cell polarity, actin filopodia formation, directional migration, and cell proliferation [153,154,155,156]. Overexpression of Cdc42 has been found in several types of cancers including lung cancer and it has been associated with tumor carcinogenesis as well as progression [157,158,159]. Additionally, accumulating evidences showed that Cdc42 plays a crucial role as metastasis regulator in human cancer models [160,161]. There is evidence to suggest that Cdc42 is involved in both the formation of invadopodia structures as well as the production and/or activation of MMPs responsible for the ECM digestion necessary for tumor cell invasion [154]. It has been documented that curcumin suppressed migration and invasion of cancer cells by downregulating Cdc42 expression in human cancer cells including lung cancer. A study conducted on human lung cancer cells A549 and 801D treated with curcumin demonstrated that curcumin downregulated Cdc-42 expression in a dose-dependent manner. The expression of Cdc42 targeting genes such as *cofilin*, *E-cadherin*, and *PAK1* also has been observed to change significantly in treated cells, suggesting that curcumin could cause anti-metastatic effects by suppressing the transcriptional level of Cdc42. Additionally, this study revealed that curcumin was able to induce rearrangement of the actin cytoskeleton as well as be involved in formation of actin filopodia, hence further strengthening the anti-metastatic activity in both A549 and 801D cells [162]. 

#### 2.3.2. Epithelial Cadherin (E-Cadherin)

E-cadherin also known as Cadherin 1 is an epithelial cell–cell adhesion molecule that functions in mediating cell–cell adhesion through calcium-dependent binding between two E-cadherin molecules at the surface of adjacent cells [163]. E-cadherin which is a transmembrane glycoprotein plays a key role in epithelial cell behaviour and cellular adhesion [164,165]. Numerous evidences have shown that the impairment and loss of function of E-cadherin were associated with cells characteristic with malignant transformation [166,167]. In addition, downregulation of E-cadherin is frequently found in tumors with extensive lymph node metastasis and infiltrative growth, suggesting that reduced level of E-cadherin contributes to cancer cell metastasis and invasion [168,169,170,171,172]. Previous study has shown that low expression of E-cadherin was detected in approximately 63% of lung carcinomas and 23% of lung adenocarcinomas [173]. 

It has been reported that curcumin is able to up-regulate the E-cadherin expression in multiple cancer cells including lung cancer, inhibiting cell invasion and metastasis. In a study with mice lung cancer, curcumin has been demonstrated to up-regulate the expression of E-cadherin through activation of heat shock protein 40 (HLJ1) also known as DNAJB4. HLJ1 activation is linked with progression of human cancer through modulation of cell proliferation, differentiation, apoptosis, invasion, and metastasis. The findings of this study also suggested that curcumin modulates HLJ1 by increasing JNK/JunD expression and subsequently reduced filopodia formation that would enhance the inhibitory activity of cell invasion and metastasis [174]. 

#### 2.3.3. Matrix Metalloproteinases (MMPs)

Matrix metalloproteinases (MMPs), a family of zinc-dependent endopeptidases play a pivotal role in proteolysis of the extracellular matrix (ECM) as these enzymes have the capability to cleave and degrade several macromolecules of ECM. In normal physiological conditions, MMPs contributes to tissue morphogenesis, organ development, wound healing, reproduction, and apoptosis, as well as angiogenesis [175]. Deregulation of MMPs has been reported to cause several pathological conditions including tumor invasion and metastasis [176,177,178]. Overexpression of MMPs particularly MMP-2 (Gelatinase A) and MMP-9 (Gelatinase B) has been associated with tumor progression, metastasis, and poor prognosis [179,180]. Numerous evidences have shown that curcumin is able to inhibit metastasis and progression of cancer cells by decreasing the expression and activity of MMPs, particularly MMP-9 and MMP-2 [181,182,183,184]. It has been reported that curcumin inhibits MMP-2 and MMP-9 expression in human NSCLC A549 cells through downregulation of the MEKK and ERK signaling pathways [185]. These protein kinases were associated with MMPs biosynthesis and have been associated with regulation of cancer cell proliferation and invasion [186,187]. This study also demonstrated that VEGF expression was downregulated indicating anti-metastatic effect in A549 cells [185]. MMP-9 may act as an angiogenic switch due to its ability to increase the bioavailability of angiogenic factors including VEGF, which is the most potent mediator of tumor vasculature [188,189]. Hence, downregulation of MMP-9 correlated with decreased expression of VEGF, resulting in inhibition of angiogenesis and metastasis. 

Another study by Chen and colleagues on human large cell lung carcinoma 801D cell line showed that the inhibitory effect of curcumin on the invasion and migration associated with decreased MMP-2 and MMP-9 expression via the inhibition of the Rac1/PAK1 signaling pathway [190]. It has been indicated that Rac1 is involved in the regulation of actin cytoskeleton rearrangement which further strengthens the anti-invasion effect of curcumin on 801D cell line [191]. In addition, curcumin has been found to reduce MMP-9 protein level in A549 cells by downregulating PKCα, NOX-2, and ATF2 expression, and inhibiting ROS intracellular production in lung cancer A549 cells. As described previously, NOX-2 is activated by PKCα which in turn promote ROS generation and subsequently activate ATF2. Activated ATF2 then facilitates AP1-binding to the MMP-9 promoter resulting in MMP-9 expression. This study demonstrated that inhibition of PKCα/NOX-2/ROS/ATF2 signaling pathway decreases expression of MMP-9, suggesting one of the mechanisms of anti-invasive effect by curcumin in lung cancer cells [192]. 

#### 2.3.4. Adiponectin 

Adiponectin is a peptide hormone produced solely by adipose tissue, which has the function to serve as an anti-atherogenic hormone by inhibiting proliferation of vascular smooth muscle cells and endothelia cells [193]. Adiponectin expression is highly associated with the risk of cancer in obesity-associated cancers such as hematologic malignancies, renal cancer, colon cancer, post-menopausal breast cancer, and endometrial cancer [194]. Whereas, low expressions of adiponectin have been detected in gastric [195] and prostate [196,197] cancers. In lung cancer, it has been reported that adiponectin is not a major predictor of risk as the expression was not significantly different compared to the control group. However, adiponectin receptors (1 and 2) that play a crucial role in adiponectin activation were expressed only in cancerous lung tissues, suggesting that adiponectin functional signaling mediates lung cancer development [198]. It has also been reported that lung cancer patients with decreased adiponectin concentration are associated with longer survival time. In addition, a significant higher adiponectin expression has been observed in NSCLC patients with metastasis compared to those without metastasis [199]. 

Tsai et al. showed that curcumin inhibits the migratory and invasive behaviour of NSCLC A549 cells. It has been shown that curcumin blocks the adiponectin receptor 1 which inhibits the adiponectin expression. Adiponectin demonstrated anti-metastatic activity by suppressing tumor angiogenesis and downregulating MMPs. This study suggested that curcumin inhibits metastasis of NSCLC cells through adiponectin/ NF-κB/MMP pathways [199].

**Figure 3:**
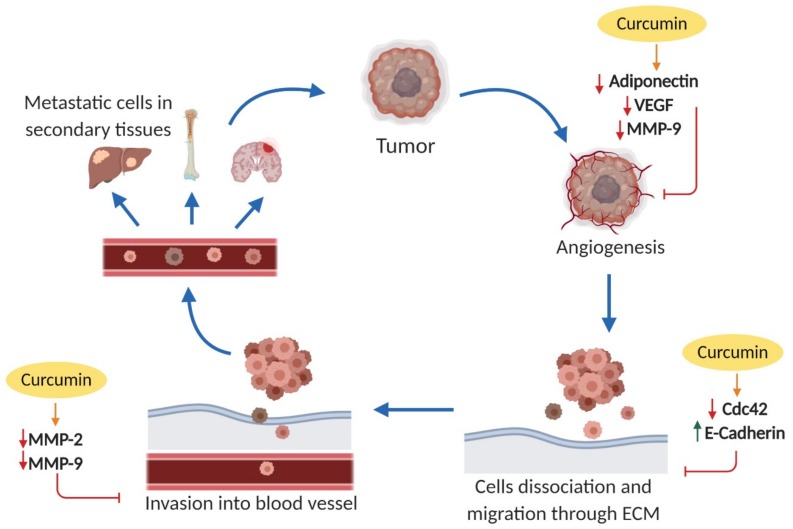
Molecular mechanism of anti-metastasis effect by curcumin against lung cancer cells. The green arrow indicates upregulation, while the red arrow indicates downregulation of molecular targets. VEGF: vascular endothelial growth factor; MMP-2: matrix metalloproteinase; MMP-9: matrix metalloproteinase-9; Cdc-42: cell division cycle 42.

Taken together, as shown in Figure 3, the findings of the studies above indicated that curcumin may act as potential anti-angiogenic and anti-metastatic agent for lung cancer cells and further research is warranted to elucidate detail mechanisms governed by curcumin in lung tumor dispersal.

### 2.4. Effects on Epigenetic Changes

Epigenetic refers to heritable changes in gene expression that occur without a change in the DNA sequence, which leads to the activation and/or silencing of multiple genes [200,201]. There are several mechanisms involve in epigenetic modification which are interconnected to selectively modulate gene expression, including DNA methylation and histone modifications [202]. Curcumin has recently been shown to induce epigenetic changes through the regulation of histone deacetylases (HDACs), histone acetyltransferases (HATs), and DNA methyltransferase 1 (DNMT1) activity that result in the activation or inactivation of the gene expression involved in cancer death and progression [203,204].

**Figure 4:**
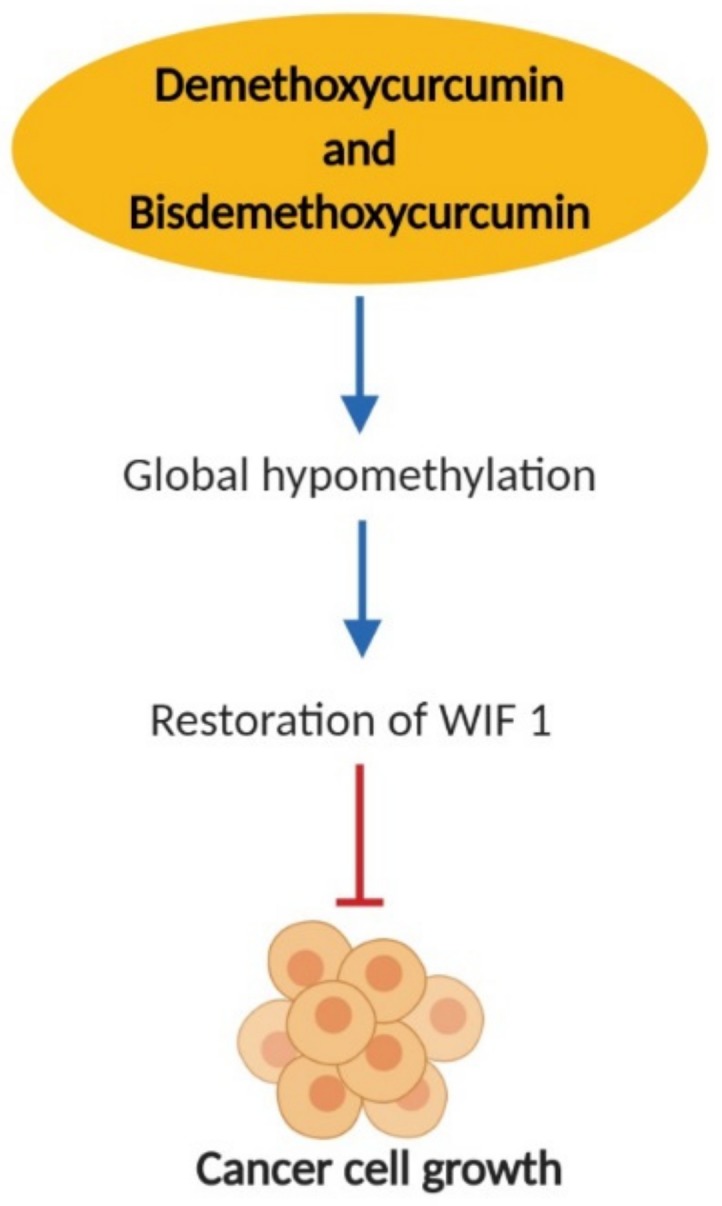
Epigenetic effect of demethoxycurcumin and bisdemethoxycurcumin on lung cancer cells. WIF 1: Wnt Inhibitory Factor-1.

DNA methylation is a covalent DNA modification that occurs mostly at 5′ position of the cytosine residues within cytosine-phosphate-guanine (CpG) dinucleotides. Moreover, it may also occur at cytosine-phosphate-adenine (CpA) and cytosine-phosphate-thymine (CpT) dinucleotides. This reaction is catalysed by DNA methyltransferase (DNMT) and S-adenosyl-methionine (SAM) as the methyl donors [205]. There are two patterns of DNA methylation that have been observed in cancer cells which include global hypomethylation and localized hypermethylation [202]. Global hypomethylation at repetitive sequences could result in genomic instability that can favor mitotic recombination followed by deletions and translocations as well as chromosomal rearrangement. This leads to activation of proto-oncogenes and pro-metastatic genes which contribute to cancer development [206,207]. Localized hypermethylation at the CpG island promoter leads to transcriptional silencing of tumor suppressor and DNA repair genes, and the inability to regulate cell cycle control, apoptosis, cell adhesion, and metastasis [208]. It has been documented that curcumin exerts anti-cancer effects against leukemia [209], cervical [210,211], melanoma [212], breast [213], and prostate [214] cancer cells through global hypomethylation which reactivates the silenced tumor suppressor and DNA repair genes thus inhibits the cancer progression [215,216]. To date, there is no study reported on curcumin as a hypomethylating agent against lung cancer cells. However, other curcuminoids, namely demethoxycurcumin and bisdemethoxycurcumin have been shown to induce demethylation effect in NSCLC cell lines A549, H460 and SPC-A-1. In this study, the demethylating effect of curcumin leads to restoration of tumor suppressor gene Wnt inhibitory factor-1 (WIF1) expression whose promoter is hypermethylated and silenced in lung cancer cells and tissues (Figure 4). As a result of WIF-1 restoration, the Wnt pathway is downregulated causing inhibition of cancer cell growth [217].

Histone modification is one of the epigenetic alterations that affects chromatin structure and function, and is subsequently involved in chromatin-based processes such as gene transcription, DNA repair, and DNA replication [218,219]. Histones (H3, H4, H2A, H2B and H1) are highly conserved core proteins of chromatin structure that subject to posttranslational modifications including lysine acetylation, ADP-ribosylation, ubiquitination, and sumoylation that occur on N- or C-terminal tail domains [220,221]. Alteration in histone acetylation has been extensively studied as it contributes to tumorigenesis [222,223,224]. Studies performed on brain cancer [225], burkitt lymphoma [226], and prostate cancer cells [227] pointed out that curcumin could be considered as a strong modulator of HDAC or HAT activity [204]. However, to date, the studies and investigations on the anti-cancer effect of curcumin in lung cancer through histone modifications remain scarce. Therefore, more investigations are warranted to elucidate the role of curcumin as a HDAC and HAT modulator in lung cancer cells.

### 2.5. The Role of MicroRNA (MiRNA)

MiRNAs are small noncoding regulatory RNAs, ranging from 19 to 25 nucleotides and are responsible to regulate gene expression at the post-transcriptional level. Furthermore, miRNAs play important roles in cell growth, proliferation, differentiation, and mobility as well as apoptosis [228,229]. Alteration in the expression of miRNAs and processing of miRNA precursors, or presence of mutations in the sequence of miRNA may have detrimental effects on cellular function and have been associated with cancer [230,231]. It has been demonstrated that miRNAs have an important role in the pathogenesis of lung cancer and development of drug-resistance [232]. A number of studies have reported an aberrant expression of miRNAs in lung tumors compared with the corresponding normal lung tissues, suggesting the involvement of miRNAs in lung cancer pathogenesis [233]. It has been described in numerous NSCLC studies that miRNAs with tumor suppressor activity are down-regulated meanwhile those with oncogenic function are upregulated [234,235,236,237]. For example, miR-34, a miRNA with tumor suppressor activity is downregulated in NSCLC cell lines. In response to DNA damage, miR-34 is activated by p53 which targets *BCL-2*, *MYC*, *MET*, and *PDGFR* genes which were associated with cell cycle regulation and apoptosis [238].

Recent evidences have shown that the pharmacological effects of curcumin in lung cancer are also mediated by modulation of several miRNAs [239]. Curcumin has been found to inhibit cancer cell growth through modulation of miRNAs such as miR-15a, miR-16, miR-21, miR-22, miR-26, miR-101, miR-146, miR-200, miR-203, and let-7, and their multiple targets genes in various types of cancer cells including lung cancer [239,240,241,242]. It has been documented that curcumin upregulated miR-16 in human lung adenocarcinoma A549 cells in which it modulates miRNA profile by upregulating eight miRNAs and downregulating six other miRNAs in which miRNA-186* is the most affected. Inhibition of miRNA-186* expression subsequently leads to upregulation of caspase-10 whereby inducing apoptosis, thus inhibiting A549 cell growth [243]. Similarly, it has been observed that curcumin downregulated miRNA-186* in cisplatin A549/DDP multidrug resistant human lung adenocarcinoma cells [244]. Taken together, these findings suggest that curcumin inhibits cell proliferation and induces apoptosis in lung cancer cells as well as drug-resistant tumor cell through downregulation of miRNA-186*. 

In addition, it has been reported that curcumin also caused a significant reduction of miR-21 expression by 60% in A549 lung cancer cells. It was indicated that the miR-21 elevates PTEN expression which inhibits cell proliferation and induces apoptosis [127]. The effect of curcumin on miRNA modulation was further demonstrated by Ye and co-workers in a study with p53 wild type H460, A427, and A549 cells. The authors showed that curcumin promoted miR-192-5p/215 upregulation, followed by activation of X-linked inhibitor of apoptosis (XIAP) signaling pathway and therefore induced apoptosis in p53 wild type lung cancer cells such as H460, A427, and A549 cells [245]. Similarly, Jin et al. also revealed that curcumin promoted upregulation of miRNA-192-5p in A549 cells, which suppresses the P13K/Akt signaling pathway. Consequently, curcumin inhibited cell proliferation and induced apoptosis in A549 NSCLC cells, as the P13K/Akt pathway plays an important role in growth factor-mediated cell survival [246].

Furthermore, curcumin has been shown to up-regulate miR-874 in A549 and H1299 cell lines which in turn targets and suppresses MMP-2 expression. It has been indicated that MMP-2 involved in the degradation of extracellular matrix facilitating the process of metastasis. Hence, downregulation of MMP-2 through upregulation of miR-874 by curcumin inhibits invasion and metastasis in A549 and H1299 cells [247]. In addition, curcumin upregulated miRNA-let7c and miR-101 in A549 cells which downregulated *EZH2* via activation of NOTCH signaling pathway. *EZH2* is an oncogene that regulates the cell cycle and progression thus downregulation was shown to inhibit proliferation of A549 cancer cells [248]. 

In a recent study, Liu et al. demonstrated that curcumin upregulates the expression of miR-98 in A549 cells followed by downregulation of LIN28A, which inhibits MMP-2 and MMP-9 activity. These findings suggested that curcumin suppressed lung cancer cell migration and invasion through activation of miRNA-98/LIN-28A/MMP-2/-9 pathways [249].

**Figure 5:**
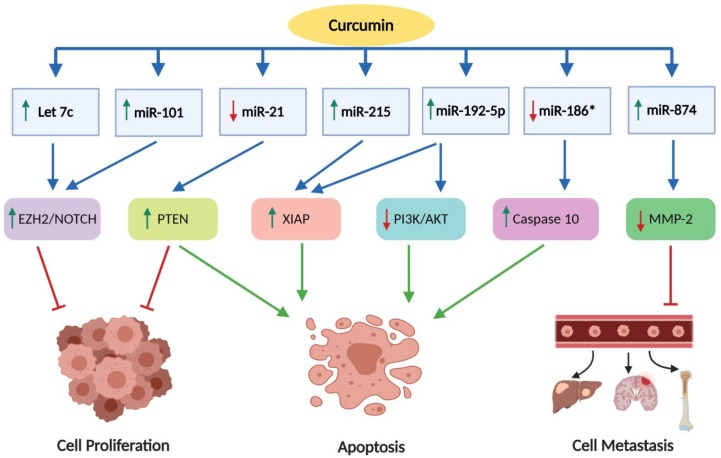
Modulation of microRNA by curcumin against lung cancer cells. The green arrow indicates upregulation, while the red arrow indicates downregulation of molecular targets and microRNAs. EZH2: enhancer of zeste homolog 2; Notch-1: neurogenic locus notch homolog protein-1; PTEN: phosphatase and tensin homolog; XIAP: X-linked inhibitor of apoptosis protein; PI3K: phosphatidylinositol 3-kinase; Akt: protein kinase; MMP-2: matrix metalloproteinase-2.

As previously described, miRNAs play an important role in the pathogenesis of lung cancer and could serve as potential therapeutic targets for lung cancer treatment. In addition, as summarized in Figure 5, all of the above findings provide an insight into the role of curcumin as potential anti-cancer agent through the modulation of miRNAs. Therefore, further investigations are crucial in this promising field to explore the link between regulation of oncogenic and tumor suppressive miRNAs and curcumin as an anti-cancer agent.

## 3. Curcumin Bioavailability Limitation and Strategies to Overcome

Pharmacokinetic profile studies of curcumin have revealed that curcumin has poor absorption as well as rapid metabolism that severely renders its bioavailability [250,251]. This is due to the metabolism of curcumin that involves glucuronidation, sulfation, and reduction which results in the formation of metabolites that have poor cell permeability and very short half-life [252]. Pan et al. reported that 99% of curcumin in plasma present as glucuronide conjugates in curcumin-treated animals. This study also showed that reduction products of curcumin such as di- and tetra-hydrocurcumin and glucuronosides of curcumin are major metabolites of curcumin in vivo [253]. These curcumin conjugates and metabolites have been revealed to play an important role in several therapeutic effects such as anti-oxidation [254], anti-inflammation [255], and anti-cancer [256]. However, multiple studies have highlighted that the anti-cancer activity by curcumin conjugates and metabolites is less potent compared to the parent curcumin [257,258,259]. Therefore, numerous strategies including improvised formulation and structural modification have been established to compensate the bioavailability limitation of curcumin [259].

Previous study by Shoba et al. have documented that the use of an adjuvant such as piperine increases curcumin bioavailability in both rats and humans by 154% and 2000% respectively via inhibition of glucuronidation. Piperine (20 mg/kg) also has ability to reduce the time duration for curcumin to achieve increased serum concentrations with no evidence of harmful results [260]. In addition, a nanoparticle of curcumin formulation has been extensively investigated to enhance the solubility and bioavailability of curcumin [261,262]. It has been found that nanocurcumin has similar effects with curcumin in reducing inflammatory responses and cell death induction against human pancreatic cancer cell lines [263]. However, the in vivo effect of this nanocurcumin is yet to be assessed. Furthermore, Ling and co-investigators revealed that a cationic liposome-PEG-PEI complex (LPPC) has been used as a carrier for the encapsulation of hydrophobic curcumin to produce curcumin/LPPC complex against 10 different cancer cells including A549 cells and LL2 mice lung carcinoma cells. This study found that the cytotoxic activity of the curcumin/LPPC was more active in 10 different cancer cells in vitro by 3.9–20 fold compared to curcumin alone. It has been suggested that the increased cytotoxic activity of curcumin/LPPC is likely attributable to its rapid accumulation in the cell [264]. It also has been reported that polymeric micellar curcumin increases the biological half-life by 60-fold in rats compared to curcumin solubilized in a mixture of PEG and dextrose [265]. Similarly, Liu et al. reported that curcumin phospholipid complex increases the elimination half-life by 1.5 fold compared to free curcumin [266]. Furthermore, curcumin analogues, and derivatives of curcumin have been widely studied in recent years. A curcumin analogue EF-24 has shown to increase absorption, produce a peak plasma level of 1000 nM, and increase the elimination half-life in mice [267]. Based on the findings mentioned above, it can be concluded that all of the approaches to counter the bioavailability issue of curcumin such as the use of adjuvants, nanoparticles of curcumin, liposomal curcumin, and analogs of curcumin may provide a new tool for cancer therapy.

## 4. Curcumin and Its Potential Side Effects

Despite several studies showed the positive biological effects of curcumin, there are studies have also highlighted the adverse effects and toxicity of curcumin intake [20,268,269]. As reported by The National Toxicology Program, there are long term as well as short term adverse effects of dietary turmeric oleoresin (79.85% similar to curcumin) in F3441N rats and B6C3F1 mice. This study was conducted by administering turmeric oleoresin in these animals at different concentrations (1000, 5000, 10,000, 25,000 or 50,000 ppm that delivers daily doses of 50, 250, 480, 1300, or 2600 mg/kg body weight) for 13 weeks and two years. In a short term (13-week) study, the toxicological signs showed an increase in liver weight, stained fur, discoloured faces, and hyperplasia of cecum and colon in both female and male rats. In addition, no sign of carcinogenic lesions and no death were observed in both female and male rats. As for a long term rats evaluation (two years), no mortality was reported while the adverse effects include incidence of ulcers, chronic inflammation, and hyperplasia of the cecum and forestomach. In addition, this study also reported the carcinogenic effects of curcumin oleoresin such as the increase in clitoral gland adenomas in female rats, hepatocellular adenomas in female mice, and intestinal carcinoma along with hepatocellular adenomas in male mice [270].

Previous study also revealed that curcumin at 25 g/kg feed significantly inhibited cyclophosphamide activity to decrease the tumor size in human breast cancer xenograft in nude mice [271]. In addition, curcumin also is proven to exhibit pro-oxidant activity, which leads to ROS generation by irreversibly modifying thioredoxin reductase and which initiates carcinogenesis [272,273,274]. It also has been reported that curcumin has the ability to cause DNA damage to mitochondrial and nuclear genomes in an in vitro study on HepG2 human hepatocellular carcinoma cells [275]. However, to date, no long term human trials with curcumin have been confirmed for its toxicity and adverse effects. However, minor adverse effects of curcumin intake in humans such as diarrhea has been reported [276].

Furthermore, early phase trials in humans have shown that curcumin can be regarded as a safe dietary supplement. As reported by Kanai and colleagues in a phase I/II human study, curcumin is safe to be consumed at doses as high as 8 g/day [277]. The Food and Drug Administration also has classified curcumin as “generally regarded as safe” (GRAS) with the panel’s conclusion that curcumin GRAS status applies for a maximal administration of 20 mg/serving or 180 mg/day of curcumin [268].

## 5. Clinical Trials 

In response to multiple in vitro and in vivo studies of curcumin in cancer cells, extensive clinical trials have been conducted on curcumin against different human cancers including pancreatic, colon, breast, cervical, and uterine [278,279]. The clinical use of curcumin in trials both as a monotherapy as well as in combination with other drugs has been shown to have anti-cancer effects, while retaining its safety [24]. Despite numerous clinical trials of curcumin in various human cancers, clinical trial of curcumin in lung cancer patients remains scarce. Therefore, more clinical trials of curcumin in lung cancer patients are needed to test its efficacy and safety as a potential therapeutic agent for lung cancer.

## 6. Conclusions

Taken together, curcumin holds a highly promising potential alternative therapy for lung cancer with less adverse effects. Curcumin exerts its anti-cancer effects in lung cancer by modulating various molecular targets, signaling pathways, epigenetics alteration, and microRNAs expression. However, the clinical application of curcumin currently is limited due to poor bioavailability, and several strategies have been evaluated to overcome this issue, thus enhancing its efficacy in lung cancer treatment. In future, a greater focus on the mechanism of curcumin by multi-omics technologies as well as clinical trials in lung cancer patients could provide more comprehensive information in understanding the therapeutic effects of curcumin against lung cancer.
